# An Overview of Tissue-Resident Memory T Cells in the Intestine: From Physiological Functions to Pathological Mechanisms

**DOI:** 10.3389/fimmu.2022.912393

**Published:** 2022-05-31

**Authors:** Yangbao Lyu, Yuming Zhou, Jun Shen

**Affiliations:** Division of Gastroenterology and Hepatology, Key Laboratory of Gastroenterology and Hepatology, Ministry of Health, Inflammatory Bowel Disease Research Center, Renji Hospital, School of Medicine, Shanghai Institute of Digestive Disease, Shanghai Jiao Tong University, Shanghai, China

**Keywords:** autoimmune disease, colorectal carcinoma, human intestine, inflammatory bowel disease, memory T cells

## Abstract

The human intestine contains a complex network of innate and adaptive immune cells that provide protective immunity. The dysfunction of this network may cause various chronic diseases. A large number of T cells in the human intestine have been identified as tissue-resident memory T cells (T_RM_). T_RM_ are present in the peripheral tissues, and they do not recirculate through the blood. It is known that T_RM_ provide rapid immune responses at the frontline of pathogen invasion. Recent evidence also suggests that these cells play a role in tumor surveillance and the pathogenesis of autoimmune diseases. In this review, we discuss the general features of intestinal T_RM_ together with their role in intestinal infection, colorectal cancer (CRC), and inflammatory bowel disease (IBD).

## Introduction

The intestinal mucosa is predominantly exposed to environmental antigens, such as food, innocuous microbes, and enteric pathogens. A complex network of innate and adaptive immune cells in the intestine mediates the protective immune responses against harmful pathogens and immune unresponsiveness to benign antigens. Among these immune cells, memory T cells provide effective and efficient immune responses to antigens previously encountered (1).

Memory T cells were primarily divided into two subsets: central memory T cells (T_CM_) and effector memory T cells (T_EM_) (2). T_CM_ express high levels of secondary lymphoid organ homing receptors CCR7 and CD62L and recirculate between the blood and secondary lymphoid organs (3). On the other hand, T_EM_ express integrins and chemokine receptors and circulate through the non-lymphoid tissues (4). With advances in technology, another lineage of memory T cells called tissue resident-memory T cells (T_RM_) (5) has been identified. Unlike T_CM_ and T_EM_, T_RM_ are permanently retained in the peripheral tissues and do not recirculate *via* the bloodstream. Apart from the location and migration patterns, they mediate local immune response to reencountered pathogens (5). In fact, most memory T cells in the intestinal mucosa possess a resident nature with distinct phenotypes and transcriptional profiles (6–8), implicating the significance of T_RM_ in intestinal immunity.

Several studies focused on the intestinal immune system have explored the potential roles of T_RM_ in pathogen defense (9), cancer immunosurveillance (10), and immunopathies (11). Understanding how T_RM_ function in intestinal immunity is vital for developing novel therapies and vaccines targeting T_RM_. Thus, this review aims to summarize the features of intestinal T_RM_ and their role in intestinal health and disease.

## T_RM_ In the Intestine

Intestinal T_RM_ are phenotypically different from T_EM_. CD69 and CD103 are the two key cell surface markers identified for T_RM_ (12, 13). Lectin CD69, a marker for early T cell activation, is reexpressed in the intestinal T_RM_, and it can be used to distinguish T_RM_ from their circulating counterparts (7). CD69 can promote T cell retention by downregulating the expression of the sphingosine-1-phosphate receptor 1 (S1PR1) protein and interacting with the transmembrane domains of the S1PR1 to suppress its function, subsequently preventing the T cells from sensing the sphingosine-1-phosphate (S1P) gradient and exiting from the gut (14–17). However, according to a recent report, the elevated CD69 expression may only serve as a passive marker rather than an essential functional regulator in driving T_RM_ cell formation in the gut (18). Another marker for T_RM_, CD103 (αE integrin), is expressed by most CD8^+^ cells and few CD4^+^ cells in the intestine (7, 12, 13, 19, 20). The αEβ7 integrin binds to E-cadherin expressed on the intestinal epithelial cells, promoting T_RM_ retention in the epithelium (21–23). However, the absence of CD103 does not rule out long-term maintenance of T cells in the intestine. Evidence showed that a small population of CD8^+^CD69^+^CD103^-^ T cells displayed persistence in the human intestine transplantation model for more than one year (12, 24). The immune repertoire of CD8^+^CD69^+^CD103^-^ T cells showed low clonal overlap with the CD8^+^CD103^+^ subset and was rather similar to T cells from peripheral blood in terms of phenotype and function (12, 24). Therefore, it can be speculated that the CD8^+^CD103^-^ T_RM_ subset represents an intermediate state between recently recruited T cells and CD8^+^CD103^+^ T_RM_. Interestingly, CD8^+^CD103^-^ T_RM_ express high levels of β2-integrin, but whether it plays a role in CD8^+^CD103^-^ T_RM_ differentiation remains unclear (24). Furthermore, a fraction of CD8^+^CD103^-^ T_RM_ express KLRG1, another ligation of E-cadherin (12, 24, 25). The comparison of CD103^+^ T_RM_ and CD103^-^ T_RM_ is summarized in **Table 1**. For CD4^+^ T_RM_ and CD8^+^ T_RM_, the phenotypical and functional differences between their CD103^+^ and CD103^-^ subsets are similar (24). In addition to CD69 and CD103, other molecules have been described as potential phenotypical markers of intestinal T_RM_, including CD49a (integrin α1 chain) (8, 12, 13), CD101 (8) and CD161 (13, 24, 28).

**Table 1:** Comparison of CD103^+^ T_RM_ and CD103^-^ T_RM_.

	CD103^+^ T_RM_	CD103^-^ T_RM_	Reference
Location	More in the IE	More in the LP	(12, 13)
Cytokine involved	TGF-β	IL-12 and TNF-β	(26, 27)
KLRG expression	None	Subset	(24, 25)
Cytotoxic	Less	More, especially the KLRG^+^ subset	(12, 25)
Cytokine release	More polyfunctional	Less polyfunctional	(24)
TCR restimulation	More responsive	Less responsive	(25)

IE, intraepithelial; LP, Lamina Propria; TGF, transforming growth factor; IL, interleukin; TNF, tumor necrosis factor; KLRG, killer cell lectin-like receptor G1; TCR, T cell receptor.

T_RM_ have a distinct pattern of transcription factor expression that coordinates T_RM_ development and survival. Hobit and Blimp-1 have been identified as two key transcription factors expressed in mice T_RM_ (29). Hobit and Blimp-1 work in synergy to repress genes required for tissue egress by binding to the S1pr1, Tcf7, and Ccr7 loci (29). Additionally, Blimp-1 alone downregulates the expression of KLF2 and subsequently S1PR1 (29). Runx3 plays a primary role in CD8^+^ T_RM_ differentiation by enhancing the expression of core residency signature and repressing the expression of signature genes of T_CM_ (30, 31). Downregulation of T-box transcription factor Eomes and T-bet is also necessary for early-stage T_RM_ differentiation (32, 33). Recent studies have found that effector-like and memory-like CD8^+^ T_RM_ subsets can be distinguished by differential expression of two transcriptional factors Blimp-1 and Id3. It was shown that Blimp-1^hi^Id3^lo^CD8^+^ T_RM_ displayed an effector gene signature and dominated the early phase of infections, whereas Blimp-1^lo^Id3^hi^CD8^+^ T_RM_ exhibited enhanced memory potential and accumulated at the late stage of infections (34).

The differentiation and maintenance of intestinal T_RM_ are highly regulated by the local microenvironment of the gut (26, 35). Upon antigen encounter, naïve CD8 T cells become activated and differentiate into short-lived effector cells (SLEC) and memory precursor cells (MPEC) (36). Early adoptive transfer experiments in mice identified the MPEC with low KLRG1 expression as the precursors of T_RM_ (21). However, a fate-mapping study on KLRG1 reporter mice revealed that more than half of the T_RM_ population in the small intestine of mice originate from the effector T cell population previously expressing KLRG1 (37). In addition, CD103^-^ T_RM_ within the intraepithelial (IE) preferentially developed from T cells that transiently expressed KLRG1 rather than those that never express KLRG1 (37). The expression of KLRG1 may be downregulated by the cytokine transforming growth factor β (TGF-β) and T cell receptor (TCR) triggering in the intestine (38). TGF-β plays a vital role in the development of the intestinal T_RM_ (39). TGF-β selectively accelerates the apoptosis of SLEC in the intestine, thus contributing to the rapid MPEC phenotype formation (26). TGF-β also downregulates KLF2 expression and subsequently downregulates the expression of S1PR1 expression (40). Additionally, TGF-β upregulates the expression of CD103 directly through Smad3 and indirectly through counteracting suppression of CD103 expression mediated by Eomes, T-bet, and TCF1 (26, 32, 41). IL-12 and TNF-β produced by inflammatory monocytes can suppress the TGF-β-induced CD103 expression, leading to the formation of CD103^-^ T_RM_ cells in the lamina propria (LP) with different transcriptional profiles (27). When exposed to extracellular ATP, the purinergic receptor P2RX7 can promote TGF-β receptor expression in CD8^+^ T_RM_ precursor, suggesting the importance of P2RX7 in T_RM_ generation (42). Recently, Peng et al. found that local ICOS signaling also promotes the generation of intestinal T_RM_ by activating the PI3K pathway and downregulating KLF2 expression (43). While the maintenance of T_CM_ and T_EM_ depends on the presence of IL-15, IL-15 is not necessary for T_RM_ retention (44).

## T_RM_ and Protection Against Pathogen

**Figure 1:**
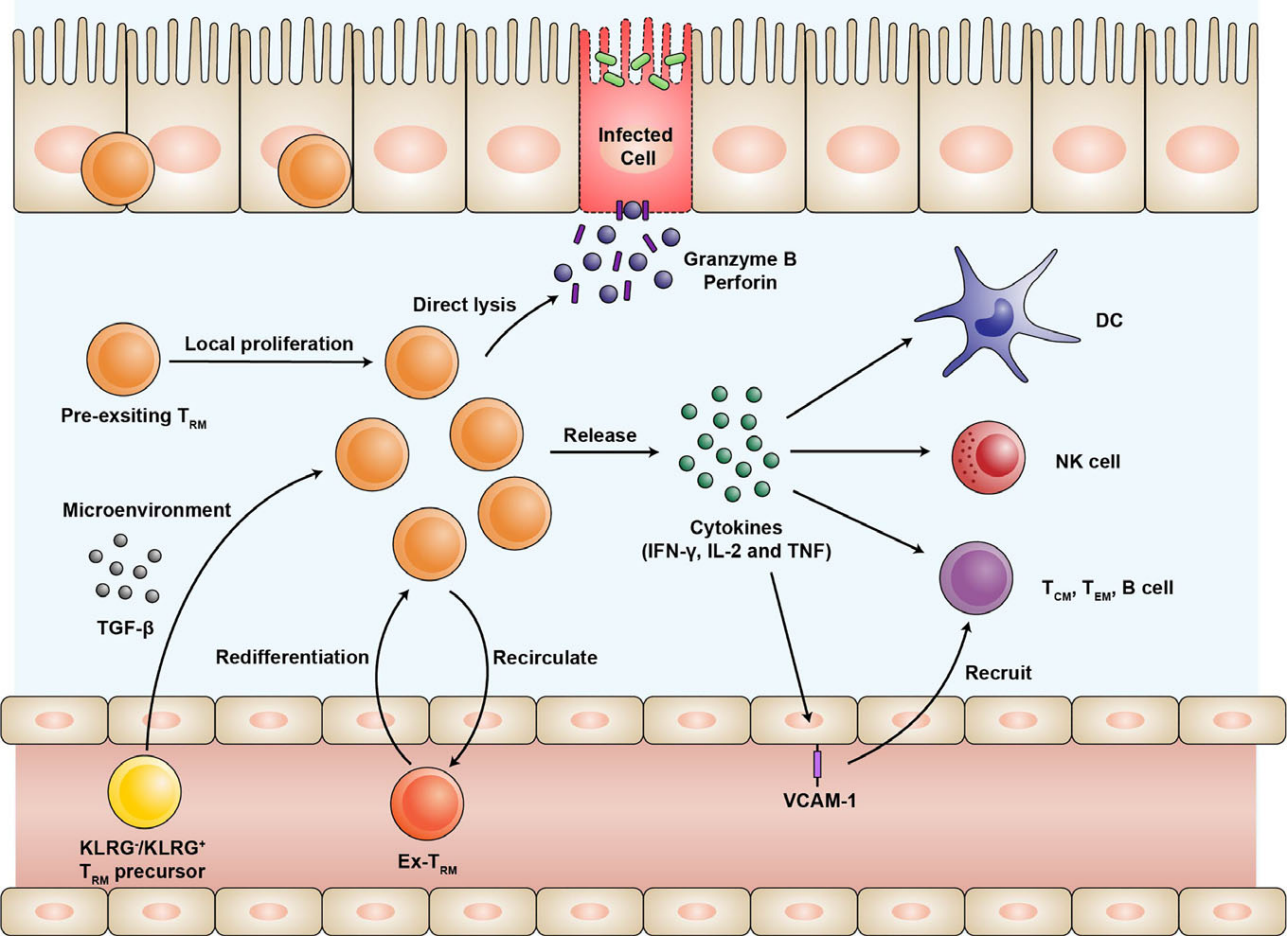
The expansion of the T_RM_ population and their protective function during infection. Effector T cells expressing KLRG or not are recruited into intestinal mucosa and differentiate into T_RM_. The differentiation of T_RM_ depends on the microenvironment of the intestine, especially TGF-β. Upon reinfection, pre-existing T_RM_ undergo local proliferation and dominate efficient recall responses. A fraction of T_RM_ may rejoin the circulation with the preference of migrating back and the potential of re-differentiating into T_RM_. T_RM_ release proinflammatory cytokines such as IFN-γ, IL-2, and TNF-α, thus activating natural killer (NK) cells and dendritic cells (DC), as well as recruiting other immune cells through upregulation of the vascular cell adhesion molecule-1 (VCAM-1) on the endothelial cells. In addition, T_RM_ can directly lyse the infected cells by producing high levels of granzyme B and perforin. T_RM_, tissue-resident memory T cells; KLRG, killer cell lectin-like receptor G1; TGF, transforming growth factor; IFN, interferon; IL, Interleukin; TNF, tumor necrosis factor; NK, natural killer; DC, dendritic cells; VCAM, vascular cell adhesion molecule.

Numerous studies have revealed that the intestinal CD4^+^ and CD8^+^ T_RM_ can be generated by intravenous or oral infections and provide enhanced regional immunity (19, 20, 26). In mice, CD4^+^ and CD8^+^ T_RM_ have been shown to provide strong protection against oral infection with *Listeria monocytogenes* (19, 26). In humans, attenuated oral typhoid vaccine can elicit activated CD4^+^ and CD8^+^ T_RM_ response (45, 46). Here, we review the two possible mechanisms by which the intestinal T_RM_ protect against pathogens rapidly and efficiently upon reinfection (**Figure 1**).

First, T_RM_ upregulate the production of proinflammatory cytokines, serving as an alarm for both innate and adaptive immune responses. Previous experiments have demonstrated the robust cytokine polyfunctionality of certain pathogen-specific CD4^+^ and CD8^+^ T_RM_ that produce high levels of interferon-γ (IFN-γ), IL-2, and tumor necrosis factor-α (TNF-α) (20, 47). The functions of T_RM_ primarily rely on the cytokines they secrete. Upon cognate peptide rechallenge, the released IFN-γ induces the expression of various antiviral and antibacterial genes in the surrounding cells to limit the initial pathogen invasion (48, 49). Furthermore, T_RM_-induced IFN-γ is required for the rapid recruitment of the circulating B and memory T cells *via* vascular cell adhesion molecule-1 (VCAM-1) upregulation on the endothelial cells (50). IL-2 stimulates granzyme B expression in the natural killer cells and the bystander memory T cells. The proinflammatory cytokine TNF-α facilitates the maturation of dendritic cells by upregulating CCR7 and other co-stimulatory molecules (49). Comparing subsets of intestinal T_RM_, LP CD8^+^CD103^+^ T_RM_ are more polyfunctional in cytokine production than LP CD8^+^CD103^-^ T_RM_ and IE CD8^+^CD103^+^ T_RM_ (12, 25). For LP CD4^+^ T_RM_, both CD103^+^ and CD103^-^ subsets are very potent producers of cytokines IFN-γ, IL-2, and TNF-α (13).

Second, some T_RM_ exhibit direct effector function. A fraction of intestinal T_RM_ can directly lyse the infected cells by expressing high levels of cytotoxic granules such as granzyme B at an early stage (23, 51). This effector-like phenotype of the intestinal T_RM_ mainly depends on the cytokine milieu of the intestinal microenvironment rather than the persistent antigen stimulation (23). Interestingly, it was found that the expression of cytotoxic granules is quite similar between CD4^+^ and CD8^+^ T_RM_ (19). CD8^+^CD103^-^ T_RM_, especially those still expressing KLRG1, exhibit high cytotoxic and proliferative potentials (12, 25). As mentioned above, they may represent T_RM_ populations recently recruited and maintained in the intestinal mucosa. Previous studies using two-photon laser scanning microscopy have shown restricted motility of the intestinal T_RM_ (52). They may stay at the sites of previous infections to surveil potential recurring pathogens. The actual contribution of the effector functions in immunologic protection mediated by the intestinal T_RM_ needs further evaluation.

As shown in the mouse infection model, intestinal T_RM_ undergo proliferation *in situ* in response to antigen rechallenging (**Figure 1**) (19, 53). Two recent studies have demonstrated that reactivated T_CM_ maintain the potential to form CD103^-^ T_RM_ and few CD103^+^ T_RM_, but the efficiency of reactivated T_CM_ is extremely lower than naïve T cells (53, 54). Under steady-state conditions, the intestinal T_RM_ populations are maintained mainly by longevity rather than local proliferation (12).

Recent evidence suggested that intestinal T_RM_ are not terminally differentiated as previously thought (**Figure 1**) (54). After pathogen rechallenge, intestinal T_RM_ are able to egress from the intestine and differentiate into circulating memory T cells. And some “ex-T_RM_” can migrate to the draining lymph nodes and give rise to the local T_RM_ (54–56). Such a process may be initiated by the antigen-driven downregulation of Hobit expression (56). In addition, “ex-T_RM_” that re-joined the circulating pool are advantaged to migrate back and re-differentiated into intestinal T_RM_ (54). A T_RM_ differentiation program is epigenetically maintained for those cells despite their developmental plasticity (54, 55). The differentiation potential of T_RM_ enables them to shape systemic T cell responses after reinfection (56).

In general, the intestinal T_RM_ can exert protective functions locally by expressing proinflammatory cytokine and cytolytic granules. Based on these findings, developing vaccines that induce T_RM_ to combat recurrent infections may be a promising strategy to enhance immunological protection.

## T_RM_ and Colorectal Cancer

CD8^+^ T cells contribute to the immunosurveillance against cancer, and their protective effects are highly correlated to their ability to enter and survive in the immunosuppressive microenvironment of the tumor compartments (10). Therefore, T_RM_ present within the tumor may be critical for controlling tumor growth. Recent reports have recognized the immunosurveillance function and prognostic significance of T_RM_ in CRC.

Over a decade ago, large numbers of CD103^+^CD8^+^ tumor-infiltrating lymphocytes (TIL) were observed in the microsatellite instability (MSI) sporadic CRC (57). Further investigations suggested that these T_RM_-like TIL may be divided into two subsets based on the expression of CD39 (58). CD39, an ectonucleotidase that hydrolyzes ATP and ADP, marks CD8 T cells for chronic antigen stimulation. The expression level of CD39 in the TIL varies significantly in the CRC patients and is remarkably high in patients with high MSI (59). CD39^+^CD103^+^ TIL are enriched for tumor antigen-specific T cells which may contribute to the control of tumor growth. In comparison, CD39^-^CD103^+^ TIL recognize cancer-unrelated epitopes and are named “bystander” T cells (59, 60). When activated *in vitro*, the CD8^+^CD39^+^CD103^+^ TIL are highly capable of killing the tumor cells in an MHC-dependent manner (59). One possible explanation is that the interaction between the CD103 on the TIL and E-cadherin on the tumor cells induces the polarization and exocytosis of cytotoxic granules at the immune synapse. As a result, the effector function of the tumor-resident memory T cells is enhanced (61). Consistent with that, a co-culture system of intestinal tumor organoids and T cells showed the significance of CD103/E-cadherin signals for antitumor immune response (62). However, whether intestinal T_RM_ are able to suppress tumors *in vivo* by target cell killing has not been validated. In addition to direct cell killing, intestinal T_RM_ may suppress tumor cell growth by secreting cytokine TNF-α and IFN-γ (63, 64). Current studies also confirmed that infiltration of CD103^+^CD8^+^ TIL is an independent predictor of survival for patients with CRC (65).

CD39^+^CD103^+^ TIL promote the gene transcription of typical markers related to exhaustion and immunomodulation (PD-1, CTLA-4, and TIM-3) (59), which implies a possible immune escape mechanism of tumor cells. Such T_RM_-like TIL are an appealing target of immune checkpoint inhibitor (ICI) therapy due to their expression of immunomodulatory markers and proliferative potential (58, 59, 66). In fact, CD103 expression highly correlates with clinical response to anti-PD-L1 immunotherapy in patients with lung and bladder cancer (67). A recent study also identified IFN-γ-producing CD8^+^ T_RM_ as a potential therapeutic target of ICI–colitis (68). It was found that most activated T cells in ICI-colitis co-express CD69 and CD103 (68). Moreover, the proportion of activated CD8^+^ T_RM_ in intestinal mucosa is highly correlated with clinical and endoscopic scores of ICI-colitis (68).

Taken together, T_RM_ contribute to the tumor immunosurveillance in the intestinal mucosa and act as a prominent target of ICI therapy and ICI-colitis. A more comprehensive understanding of the identification, regulation, and function of T_RM_-like TIL is required for improving current cancer treatment.

## T_RM_ and Inflammatory Bowel Disease

IBD, including Crohn’s disease (CD) and ulcerative colitis (UC), is a chronic inflammatory disease of the intestinal tract characterized by local relapsing flares (69). The pathogenesis of IBD involves abnormal immune responses against intestinal microbiome (70). Some properties of T_RM_ suggest that they may be the key player in the onset and relapse of IBD. T_RM_ possess the capacity to secrete pro-inflammatory cytokine and recruit other leukocytes. Also, T_RM_ in the intestine mucosa are confined to certain regions with limited ability to migrate, which may explain the localized pattern of flares seen in IBD (52).

Recent evidence suggests that CD4^+^ T_RM_ may serve as a driver for IBD (71). Zundler et al. reported that knockout of Hobit and Blimp-1, the two core transcriptional factors of T_RM_, prevented the development of colitis in several experimental mouse models (71). Furthermore, in the T cell transfer colitis model, the mouse with Hobit-Blimp-1 double knockout CD4^+^ T cells showed impaired secretion of pro-inflammatory cytokines (IFN-γ, IL-13, and IL-17A) and recruitment of granulocytes and macrophages (71). In humans, the same authors observed that CD4^+^CD69^+^ T cells in the LP of IBD patients produce markedly more pro-inflammatory cytokines (IFN-γ, IL-13, IL-17A, and TNF) when compared to CD4^+^CD69^-^ T cells. High CD4^+^CD69^+^CD103^+^ T_RM_ proportion in LP was prospectively associated with earlier relapse in both UC and CD patients (71). Consistently, a study by Lamb et al. showed that the high expression of CD103 in CD4^+^ T cells is associated with elevated pro-inflammatory cytokine production and lowered regulatory markers expression in UC patients (72). Furthermore, CD4^+^CD103^+^ T_RM_ are enriched for Th17/Th1 lineage cells, which co-express IL-17A and IFN-γ (72). Bishu et al. investigated colon samples of CD patients and found that CD4^+^ T_RM_ in CD patients expresses higher levels of IFN-γ and IL-17A relative to controls (73). Particularly, CD4^+^ T_RM_ are identified as the major mucosal TNF-α-producing T cell subsets in the CD patients (73). Despite the pathogenic role of CD4^+^ T_RM,_ it remains controversial whether CD4^+^ CD103^+^ T_RM_ are increased in the gut of IBD patients (71, 74).

The heterogeneity and functional profile of CD8^+^ T_RM_ in IBD were also investigated. Bottois et al. observed two distinct subsets of CD8^+^ T_RM_ in the ileum of CD patients, defined by the mutually exclusive expression of CD103 and KLRG1 (25). CD8^+^ CD103^+^ T_RM_ were decreased in inflamed mucosa from IBD patients when compared to non-inflamed mucosa and controls, while CD8^+^KLRG1^+^ T_RM_ showed a significant increase (25). CD8^+^CD103^+^ T_RM_ in CD patients expressed higher levels of IL22, IL26, and CCL20. These three Th17-related cytokines are involved in tissue homeostasis and innate immune response (25). Similarly, single-cell RNA-sequencing of CD8^+^ T cells from the colon of UC patients showed that the expression of IL26 was enriched in the CD8^+^CD103^+^ population (75). In addition, CD8^+^CD103^+^ T_RM_ express high levels of CD39 and CD73, two key functional markers of regulatory T cells (76). CD39 and CD73 can hydrolyze extracellular ATP into adenosine, which is a potent immunoregulator (77). Roosenboom et al. observed that CD8^+^CD103^+^ T_RM_ were significantly decreased in patients with active IBD compared to patients with endoscopic remission and healthy controls (74). These findings suggest that CD8^+^CD103^+^ T_RM_ may contribute to tissue homeostasis and immunoregulation. By contrast, CD8^+^KLRG^+^ T_RM_ have higher proliferative and cytotoxic potential (12, 25).

The pathogenic role of T_RM_ makes them a promising target for IBD treatment. For instance, etrolizumab is a humanized monoclonal antibody that targets the β7 subunits of αEβ7 and α4β7 integrins. In a completed phase III trial in UC, etrolizumab met its primary endpoint in two induction studies but not in any maintenance studies (78–81). Phase III trials assessing etrolizumab in CD are still ongoing with positive results in an exploratory induction cohort (82). The clinical efficacy of Etrolizumab may be partially explained by impairing the retention of CD103^+^ T_RM_. In fact, a phase II trial in UC showed that the response to etrolizumab is associated with the number of CD103^+^ cell in the colon samples (83). However, CD103 is also expressed on a subset of intestinal dendritic cells (84), so it remains to be investigated whether T_RM_ are the real target of etrolizumab. Another pharmacological that might target T_RM_ is S1PR modulators. Recently, the S1PR modulator ozanimod has been approved for UC (85). By triggering the internalization and degradation of the S1P receptors, S1PR modulators inhibit lymphocyte egress from lymphoid organs and may impair local generation of intestinal T_RM_ (86).

Collectively, a growing body of evidence implicates that T_RM_ participate in the pathogenesis of IBD. Therefore, they might be the potential target of some currently developed drugs, especially etrolizumab and S1PR modulators. It is not surprising that T_RM_ have also been identified as key mediators in other intestinal immunopathologies, such as celiac disease (87) and acute graft-versus-host disease (88). Further exploration is needed to better understand the role T_RM_ play in different intestinal immunopathologies and to develop therapies that specifically target pathogenic T_RM_.

## Conclusions

A large number of CD8+ and CD4+ T_RM_ are permanently located in the human intestinal LP and IE (12, 13). After developing within the gut microenvironment, CD8+ and CD4+ T_RM_ differ from their circulating counterpart both at the phenotypical level and transcriptional level (29). In recent years, studies in mouse models and human biopsies have demonstrated a crucial role for intestine T_RM_ in the protection against enteric pathogens, immunosurveillance of intestinal malignancy, and probably inducing intestinal chronic inflammation (26, 59, 71). Advancing our knowledge of the properties and functions of intestinal T_RM_ may provide insights into developing efficacious vaccines and therapies against intestinal diseases.

## Author Contributions

YL and YZ collected the papers and data, analyzed the conclusions, and drafted the manuscript; JS presented the idea of this paper, supported the funding, analyzed the conclusions, drafted and revised the manuscript. All authors contributed to the article and approved the submitted version.

## Funding

Supported by grants from Shanghai Science and Technology Innovation Initiative (21SQBS02302), and Cultivated Funding for Clinical Research Innovation, Renji Hospital, School of Medicine, Shanghai Jiaotong University (RJPY-LX-004).

## Conflict of Interest

The authors declare that the research was conducted in the absence of any commercial or financial relationships that could be construed as a potential conflict of interest.

## Publisher’s Note

All claims expressed in this article are solely those of the authors and do not necessarily represent those of their affiliated organizations, or those of the publisher, the editors and the reviewers. Any product that may be evaluated in this article, or claim that may be made by its manufacturer, is not guaranteed or endorsed by the publisher.
